# High Resolution Consensus Mapping of Quantitative Trait Loci for Fiber Strength, Length and Micronaire on Chromosome 25 of the Upland Cotton (*Gossypium hirsutum* L.)

**DOI:** 10.1371/journal.pone.0135430

**Published:** 2015-08-11

**Authors:** Zhen Zhang, Junwen Li, Jamshed Muhammad, Juan Cai, Fei Jia, Yuzhen Shi, Juwu Gong, Haihong Shang, Aiying Liu, Tingting Chen, Qun Ge, Koffi Kibalou Palanga, Quanwei Lu, Xiaoying Deng, Yunna Tan, Wei Li, Linyang Sun, Wankui Gong, Youlu Yuan

**Affiliations:** 1 State Key Laboratory of Cotton Biology, Key Laboratory of biological and genetic breeding of cotton, The Ministry of Agriculture, Institute of Cotton Research, Chinese Academy of Agricultural Sciences, Anyang, 455000, Henan, China; 2 Anyang Collage of Technology, Anyang, 455000, Henan, China; USDA-ARS-SRRC, UNITED STATES

## Abstract

Cotton (*Gossypium hirsutum* L.) is an important agricultural crop that provides renewable natural fiber resources for the global textile industry. Technological developments in the textile industry and improvements in human living standards have increased the requirement for supplies and better quality cotton. Upland cotton 0–153 is an elite cultivar harboring strong fiber strength genes. To conduct quantitative trait locus (QTL) mapping for fiber quality in 0–153, we developed a population of 196 recombinant inbred lines (RILs) from a cross between 0–153 and sGK9708. The fiber quality traits in 11 environments were measured and a genetic linkage map of chromosome 25 comprising 210 loci was constructed using this RIL population, mainly using simple sequence repeat markers and single nucleotide polymorphism markers. QTLs were identified across diverse environments using the composite interval mapping method. A total of 37 QTLs for fiber quality traits were identified on chromosome 25, of which 17 were stably expressed in at least in two environments. A stable fiber strength QTL, *qFS-chr25-4*, which was detected in seven environments and was located in the marker interval between CRI-SNP120491 and BNL2572, could explain 6.53%–11.83% of the observed phenotypic variations. Meta-analysis also confirmed the above QTLs with previous reports. Application of these QTLs could contribute to improving fiber quality and provide information for marker-assisted selection.

## Introduction

Cotton is an important agricultural crop around the world, providing a renewable natural fiber source for the textile industry. Among the four species of cotton family, upland cotton (*Gossypium hirsutum* L., 2n = 52) is the most widely planted for its high yield potential and attractive fiber quality. Since hybridization breeding was initiated in plants, significant achievements have been attained in cotton improvement. However, innovations in textile technology and improvements in living standards continuously occur, thus increasing the requirement in cotton fiber yield and quality. The yield and fiber quality are quantitative traits and there is a negative genetic correlation between fiber quality and yield; therefore, traditional breeding methods based on phenotypic selection become increasingly difficult [1,2,3].

Marker-assisted selection (MAS) is a relatively recent innovation in breeding for crop improvement. Molecular genetic markers are developed based on DNA variations among individuals in a breeding population. In contrast to phenotypic selection, MAS is a direct form of genotype selection. Theoretically, MAS could be applied to quantitative trait locus (QTL) selection, as well as breaking the negative correlation between the target traits [4]. However, efficient MAS require a marker-saturated genetic map with high resolution. Therefore, many efforts have been made in genetic mapping and QTL identification in cotton using temporary [1,2,5,6,7,8,9,10,11,12] and/or permanent populations [13,14,15,16,17]. Wu et al. [16] identified six QTLs, accounting for 56.90% of the observed phenotypic variations (PV) in fiber strength (FS) and five QTLs responsible for 38.10% of the observed PV in lint percentage. Liang et al. [18] identified 39 QTLs for fiber quality traits in four environments in separate analyses. Ning et al. [19] discovered 23 QTLs for fiber strength distributed on 10 chromosomes that explained 3.73%–17.55% of the observed PV, with logarithm of odds (LOD) scores ranging from 2.86 to 7.09 and thirteen QTLs for seed cotton yield on eight chromosomes, each explaining 5.86–30.61% of the observed PV, with LOD scores ranging from 2.91 to 7.83. Shao et al.[20] identified 77 QTLs for five fiber quality traits in three populations, including 46 significant QTLs and 31 putative QTLs, which were mapped to 24 chromosomes. Applications of these findings could result in significant improvements in cotton breeding efficiency and shorten the breeding cycle.

However, there is a considerable distance between QTL identification and its pragmatic application in breeding programs, such as: (1) mapping of markers has not reached saturation, especially for markers that are based on intraspecific mapping populations, and there are large marker gaps in the QTL regions [18,20,21,22] that could negatively affect marker based selection and reduce selection efficiency. (2) Furthermore, these mapping efforts are still not based on the whole genome [18,21,22], which makes it difficult to obtain a complete picture of the QTLs for the target trait and also reduces selection efficiency.

In our laboratory, a map was constructed with using a recombinant inbred line (RIL) population developed from a cross between upland cotton 0–153 and sGK9708. Using this map, we identified two major QTLs for fiber strength, two QTLs for fiber length (upper half mean length, FL), and one QTL for fiber micronaire (FM) on chromosome 25 (chr25) [23]. These results suggested that chr25 could be an important chromosome harboring fiber quality QTLs. However, the linkage map has a total distance of 62.20 centimorgans (cM) and only 23 markers were screened and mapped, with an average marker distance of about 2.70 cM and marker gaps larger than 10 cM. This problem exists in nearly in all report we examined [20, 24, 25]. Using interspecific crosses between *G*. *hirsutum* and *G*. *barbadense*, Yu et al. [24] identified two FM QTLs from F_2_ population and two FM QTLs from the testcross population. Yang et al. [23] identified two FM QTLs from a backcross of one population (BC_1_). Shao et al. [20] identified two intraspecific fiber strength QTLs and one micronaire QTL of *G*. *hirsutum*. All of the maps used in QTL mapping were neither integrative nor saturated, and the position and range of the mapped QTLs were neither precise nor accurate.

Chr25 is regarded as chromosome 6 of the D-subgenome. Although the D-genome species of *Gossypium* do not produce spinnable fiber and the genome size of the D-genome is only half of that of the A-genome, it is estimated that both bothhave same total numbers of genes [26, 27, 28]. After an allopolyploidization event between the A-genome and the D-genome, the formed tetraploid upland cotton markedly changed its plant morphology to economically significant properties, suggesting that the D-subgenome may also contribute specific to fiber quality traits. Therefore, we decided to construct a high-resolution linkage map of the QTLs on chr25 and assess their stability across multiple environments, based on the study conducted by Sun et al. [23]. A high-resolution map that harbors more markers, including simple sequence repeat (SSR) and single nucleotide polymorphism (SNP) markers, without any large gaps, especially in the QTL positions, would increase the accuracy of the QTLs and aid in the selection of markers in subsequent breeding programs.

## Materials and Methods

### Plant materials

**Fig 1:**
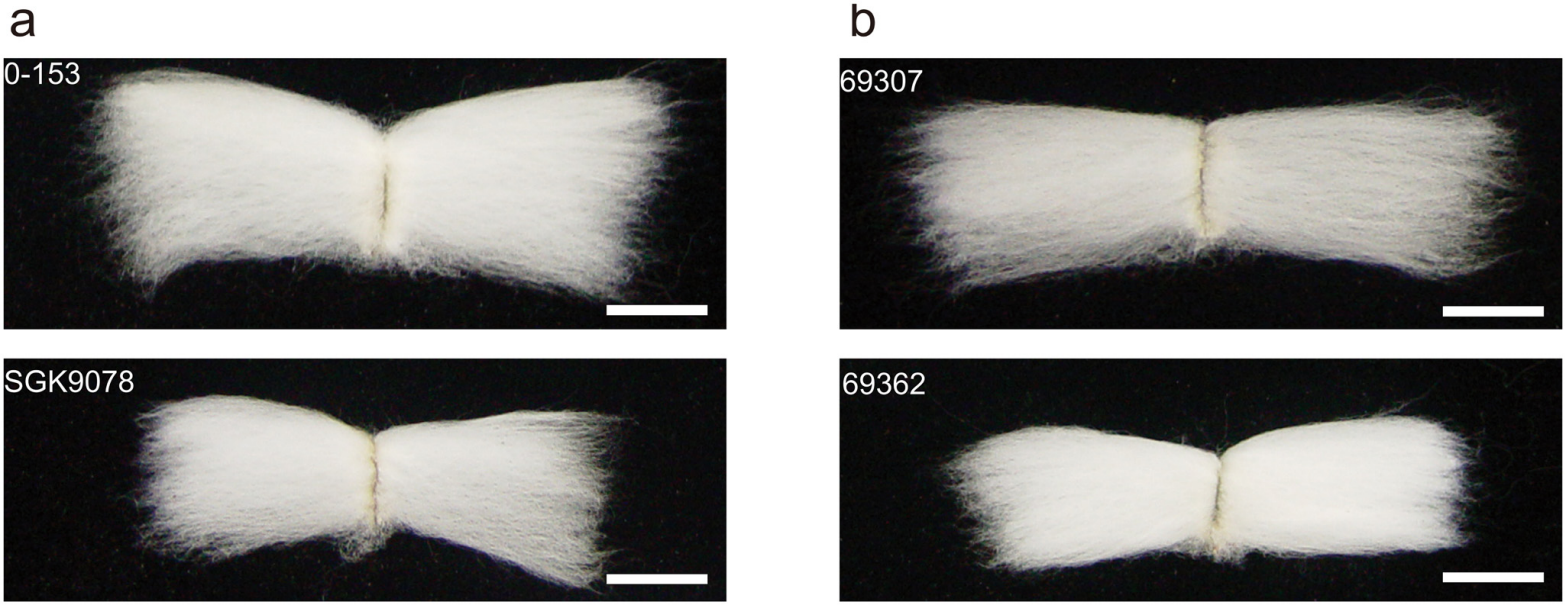
Phenotypic differences of fibers among the parents and the typical lines in the RIL population. a: Phenotypes of fibers of 0–153 and sGK9708; b: Phenotypes of fibers of two typical lines (69307 and 69362). The scale bar indicates 1 cm.

F_6:8_ recombinant inbred lines with 196 plant lines were developed from the intraspecific cross of two upland cotton cultivars, sGK9708, derived from CRI41, which is a widely planted cultivar with high yield and wide adaptability, and 0–153, an elite upland cotton cultivar harboring strong fiber strength genes (Fig 1). The detailed information on the development of the RIL population was earlier described by Sun et al [23]. For convenience in description and research, the F_6:8_ and the next generations derived from it were all regarded as F_6:8_.

From 2007 to 2013, multiple environmental evaluations in 11 environments were conducted at six ecological locations. In 2007, one evaluation was conducted in Anyang of the Henan Province; in 2008, three were conducted in Anyang of Henan Province, Linqing in the Shandong Province and Quzhou in the Hebei Province; in 2009, three were conducted in Anyang of the Henan Province, Quzhou of the Hebei Province and Akesu in the Xinjiang Autonomous Region; in 2010, three were conducted in Gaoyi of the Hebei Province, and Anyang and Zhengzhou of the Henan Province, and in 2013, one was conducted in Anyang of the Henan Province (all11 environments were hereinafter referred to as 07ay, 08ay, 08lq, 08qz, 09ay, 09qz, 09aks, 10gy, 10ay 10zz and 13ay, respectively). A randomized incomplete-block design with two replicate was adopted in the 11 environmental evaluations. The details of the field evaluations from 2007 to 2008 were same as in Sun’s report [23]. Single-row plots, with 0.8-m row distances and 5-m row lengths were adopted in 09ay, 10ay, 10zz, and 13ay, whereas, single-row plots, with 0.8/0.6-m row distances and 5-m row lengths were adopted in 09qz and 10gy; and six-row plots, with 0.6/0.1-m row distances and 2-m row lengths were adopted in 09aks. The field evaluation of 08lq was conducted in the experimental field and approved by the Cotton Research Center of Shandong Province. The field evaluations of 08qz and 09qz were conducted in the experimental field and approved by the Ecological Station of Agricultural University of China. All other field evaluations were conducted in the experimental field of the Ecological Station of the Institute of Cotton Research, Chinese Academy of Agricultural Sciences (CAAS).

### Phenotypic data analysis

In September of each year, 30 normally opened bolls in the plots were harvested to their test fiber quality using an HVI1000 (Uster Technologies, Switzerland) with HVICC Calibration in the Cotton Quality Supervision, Inspection and Testing Center, Ministry of Agriculture, Anyang, China. The fiber quality traits include fiber length, fiber strength and micronaire. The basic statistics of the phenotypic data of the population such as mean value, standard deviation and skewness value of the fiber quality traits were processed by Microsoft Excel 2010. The significance of differences in fiber quality traits between the two parents was evaluated by one-way ANOVA using Microsoft Excel 2010. Pearson correlation coefficients by using the software SPSS20.0 were calculate to determine the degree of association among traits.

### Analysis of SSR markers

Genomic DNA of the two parents, F_1_ and 196 F_6:8_ RILs were extracted using the cetyl trimethylammonium bromide (CTAB) method [29]. A total of 7,300 SSR primer pairs were used to screen for polymorphisms between the parents. These primers included 1,000 pairs designed based on the D genome and 1,000 based on the A genome of chr25 and its homologous chromosome. The other primer pairs used in this research are available at the Cotton Marker Database (CMD; http://www.cottonmarkerdatabase.org). These primers were synthesized by Shanghai Invitrogen Company (Shanghai, China) and Shenzhen BGI Company (Shengzhen, China). All the polymorphic primer pairs were used to genotype the RIL population, F_1_ and the parents. PCR was conducted as described by Sun et al. [23] and electrophoresis and detection of PCR products were conducted according to Zhang [30]. The sequence information of the new SSR markers is listed in S1 Table.

### Analysis of SNP markers

Bulked segregation analysis (BSA): BSA was used to group the RIL population and its parents, which generated four pools. The four pools included two parent pools, one pool for strong fiber strength comprising 25 lines with the strongest fiber strength among the 196 RILs, and one for weak fiber strength consisting of 25 lines with the weakest fiber strength.

Specific-locus amplified fragment sequencing (SLAF-seq): SLAF was performed according to Sun et al. [31] with minor modifications. First, genomic DNA was incubated at 37°C. The PCR reaction was performed using diluted restriction-ligation samples, dNTP, Taq DNA polymerase (NEB, NE, USA) and an *MseI* primer containing barcode1. The PCR products were purified and then pooled. The pooled samples were incubated with *Mse*I, T4-DNA ligase, ATP, and a Solexa adapter at 37°C and then purified. The purified products were run on a 2% agarose gel. Fragments of 500–550 base pairs (bp) (with indices and adaptors) in size were isolated. The fragment products were subjected to PCR with Phusion Master Mix (NEB) and Solexa amplification primer mix to add barcode2. Products of 500–550 bp in size were gel-purified and diluted for pair-end sequencing (PES). PES was performed using an Illumina high-throughput sequencing platform (Illumina, Inc., San Diego, CA, USA).

SLAF-seq data grouping and genotyping: All SLAF single-end reads of the PES were grouped based on sequence similarity, as detected by BLAT [32] (-tile size = 11, -step size = 11). Sequences with 90% similarity were clustered into one SLAF locus. Alleles were designated as specific to each SLAF according to minor allele frequency. Because cotton is a tetraploid, there are at most eight kinds of SLAF tags in one locus. Threrefore, clusters with more than eight tags were regarded as repetitive SLAFs, and were filtered out. SLAFs with more than one tag were considered polymorphic SLAFs and regarded as potential markers. Potential markers were classified into three segregation patterns. We verified the origin of alleles according to the sequencing depth of the parent. Potential markers with one genotype derived from the paternal and the other from the maternal were identified as markers.

Allele specific primer-polymerase chain reaction (ASP-PCR) [33, 34]: ASP-PCR was used to detect the above mentioned SLAF markers across the entire RIL population. Based on the sequence of the SNP in the SLAFs, two ASP primers were designed with each of the SNP bases as its 3′ end according while introducing one mutant at the -1 to -3 position of the 3′ end of the primers. ASCP primers were completely designed according the DNA sequence of the ASP primers [35, 36, 37]. The software Primer Premier 5 was used to design all primers. PCR was conducted in total volume of 10μL, with 30 ng–40 ng of cotton DNA, 1.0μL 10×PCR Buffer (Mg2+ plus), 0.5μL 10Mm of dNTPs, 1μL of each primer, and 0.5 units of Taq (Dalian TaKaRa Company, Dalian, China). The PCR conditions were as follows: 95°C for 3 min; 30 cycles of 94°C for 30 s, 50°C–65°C (according to the melting temperature of the primers) for 30 s, and 74°C for 1 min; after that, 72°C for 5 min. PCR products were then subjected to agarose gel electrophoresis. For screening of each of the SNP marker, ASP-PCR was conducted in pairs using the ASP1+ASCP and ASP2+ASCP primers, respectively. The combination of ASP1+ASCP-producing and ASP2+ASCP-not producing target band, or ASP1+ASCP-not producing and ASP2+ASCP-producing target band was indicative of the presence of a homozygote in one of the dimorphs of the SNP. when both ASP1+ASCP and ASP2+ASCP produce the target band, this was considered indicative that the SNP position was heterozygous. The SNP markers developed were designated as CRI-SNP. The sequence information of the SNP markers is presented in S2 Table.

### Linkage map construction

JoinMap 4.0 Version Software [38] was used for linkage analysis with the log of odds (LOD) score set at 6.0 and a maximum genetic distance of 50 (cM) for the RIL population. The Kosambi mapping function [39] was used to convert recombination frequencies into map distances. A graphical representation of the linkage groups was created by using Map Chart 2.2 [40].

### QTL mapping

To identify QTLs for fiber quality traits, RIL linkage maps and RIL population data of fiber quality traits in 11 environments were used. QTLs were analyzed by using the composite interval mapping method (CIM) [41] as provided in Windows QTL Cartographer 2.5 [42]. The LOD threshold for declaring a significant QTL was calculated by using a mapping step of 1.0 cM, five control markers, and 1,000 permutation tests at significance level of P < 0.05.. LOD scores between 2.0 and permutation test LOD threshold were used to declare suggestive QTLs. QTLs for the same trait across different environments were considered stable QTLs and expressed across multi-environments when their confidence intervals overlapped.

The QTLs were named as follows: (q + trait abbreviation) + chromosome/ + QTL number.

### Meta-analysis of QTLs

The Biomercator V4.2 software [43, 40] was used to construct a consensus map using data from previous reports [20, 23] and the map in the present study and to identify QTL hot spots and clusters. Two input files, a map file and a QTL file, were prepared for the Biomercator V4.2 software according to its requirements. Four models each with an Akaike information criterion (AIC) value were provided by the software. The model with the lowest AIC value was used to predict the most probable meta-QTL (mQTL). The mQTLs on the consensus map aggregate approximately within less than 10 cM regions. Therefore, QTL clusters were manually defined as a QTL-rich region that contained five or more QTLs of various trait types, and hotspots were manually defined as five or more QTLs of the same trait type within a 10cM region [44, 45]

### Expression of candidate genes linked to specific QTL markers

Candidate genes, namely Gh_D06G0694, Gh_D06G0721, Gh_D06G0740 and Gh_D06G1025, which were linked to qFS-chr25-6, qFL-chr25-4, qFS-chr25-4 and qFL-chr25-3 respectively, were selected from Zhang et al [46] to conduct Expression verification. Two lines from the 0–153×sGK9708 population, 69307 with longer and stronger cotton fiber and 69362 with shorter and weaker cotton fiber (Fig 1), were used for candidate gene expression. RNA samples of lint were extracted from the lint of balls of 10, 15, 20, 25, 30 dpa, of newly uncurled leafs at seedling stage, and of cotyledon, root, hypocotyl and stem were extracted from seedlings incubated in a growth chamber at 30°C and a photoperiod of 14-h illumination. Plant total RNA was extracted using the RNA prep Pure Plant Kit (polysaccharide- and polyphenolics-rich) of Tian Gen Company (Beijing, China), following the manufacturer’ protocol. Expression was performed by using the PrimeScript RT reagent Kit with gDNA Eraser (Perfect Real Time) of Takara Bio Inc (Dalian China), following the manufacturer’s instructions Briefly, gDNA was erased from the total RNA samples with a gDNA eraser. Then, reverse transcription-polymerase chain reaction (RT-PCR) was conducted using the gDNA free RNA samples. Real-time quantitative PCR (RT-qPCR) was performed by using SYBR premix Ex Taq (Tli RNaseH plus) (Takara, Dalian China). RT-qPCR was performed on an Applied Biosystems 7500 fast Real Time PCR system (NY, USA) using following protocol: one cycle at 95°C for 30 s; followed by 40 cycles at 95°C for 5 s and 60°C for 34 s; and finally one cycle at 72°C for 30 s. The primers used in the RT-qPCR assays were designed using software Primer Premier V with the highest rating values.

## Results

### Trait performance in the RIL populations

The descriptive statistics of the fiber quality traits, namely fiber strength, fiber length, and fiber micronaire, as well as the performance of the RIL populations and their parents are shown in Table 1. All three traits showed normal distributions in their mean values. Strain 0–153 demonstrated significantly greater fiber strength and fiber length values than those of sGK9708, except for the FM value, for which no significant difference was detected between 0–153 and sGK9708. Correlation analysis indicated that fiber strength and fiber length were significantly positively correlated whereas fiber strength and FM, and fiber length and FM were significantly negatively correlated (S3 Table).

**Table 1:** Statistics of the mean phenotypic values of fiber length, fiber micronaire and fiber strength of RILs and their parents in 11 environments.

Trait	Parents				RILs				
	0–153 (P1)	sGK9708 (P2)	P1-P2	P-value	Range	Mean	SD	Kurtosis	skewness
FL	30.25	27.39	2.86	<0.001***	26.26–32.36	29.19	1.27	-0.34	-0.10
FM	4.41	4.80	-0.38	0.139	3.12–5.21	4.37	0.39	0.03	-0.21
FS	33.27	25.75	7.51	<0.001***	25.74–36.10	30.04	0.87	0.27	0.31

*** indicates significance at P ≤ 0.001.

### Constituents and screening of markers

Two types of markers were screened and mapped onto chr25 in the present study: SSRs and SNPs. A total of 7300 SSR primer pairs were screened for polymorphisms between the parents 0–153 and sGK9708, 97 (1.32%) of which generated polymorphic results that were related to chr25. Among the 97 polymorphic SSR primer pairs, four pairs produced smear bands across the RIL population and could not be used in the construction of linkage relationships whereas the remaining 93 SSR primer pairs from 20 sources produced 106 polymorphic loci that could be used in linkage map construction. The names, numbers, percentage and sources of the 93 pairs are listed in Table 2, among which the SWU SSR primer pairs designed by the Southwest University of China were developed on the basis of the D genome sequence reference. The SWU SSR marker produces 31 polymorphic primer pairs, the NAU SSR marker ten, the DPL SSR marker nine, and the BNL SSR marker eight, whereas most of the primers from other sources produce only 1–2 (Table 2); the percentage of polymorphic primer pairs among the total primer pairs screened was very low [23].

**Table 2:** The information of the SSR markers and SNP markers on the linkage map.

Primer Name	Marker Type	Number of polymorphism primers	Percent of polymorphism primers	Discovered by
CRI-SNP	SNP	104	52.79%	Institute Of Cotton Research of CAAS, CHN
BNL	SSR	8	4.06%	Brookhaven National Laboratory, NY
CGR	SSR	6	3.05%	Monsanto company, USA
CIR	SSR	1	0.51%	CIRAD, France
CM	SSR	1	0.51%	Texas A&M University, USA
COT	SSR	2	1.02%	Monsanto Company, USA
DC	SSR	1	0.51%	Monsanto Company, USA
DOW	SSR	1	0.51%	DOW agricultural science, India
DPL	SSR	9	4.57%	Monsanto Company, USA
GH	SSR	4	2.03%	Texas A&M University, USA
HAU	SSR	5	2.54%	Huazhong Agricultural University. CHN
ICRI	SSR	3	1.52%	Institute Of Cotton Research of CAAS, CHN
JESPR	SSR	2	1.02%	Texas A&M University, USA
NAU	SSR	10	5.08%	Nanjing Agricultural University, CHN
NBRI	SSR	2	1.02%	National Botanical Research Insitate, India
PGML	SSR	2	1.02%	Southwest University, CHN
SHH	SSR	2	1.02%	Institute Of Cotton Research of CAAS, CHN
SHIN	SSR	1	0.51%	Monsanto Company, USA
SWU	SSR	31	15.74%	Southwest University, CHN
TMB	SSR	1	0.51%	USDA-ARS, Texas
TMK	SSR	1	0.51%	USDA-ARS, Texas
Total	SSR	197	100%	

Among the SNP markers, 104 markers were developed that mapped to chr25 using BSA screening [28]. Unlike the positions of the SSR markers, which were randomly interspersed across in the genome, the identified SNP loci could be mapped and their positions were almost evenly distributed across the linkage group. There were also in four positions in the linkage group where the SSR and SNP markers were tightly linked and segregated as bins, namely, CRI-SNP145524 and HAU1355; CRI-SNP120491 and NAU2119; CRI-SNP153258 and DC40429; and BNL3806a and TMK19 (Table 2 and S1 Fig).

### Construction of linkage maps and collinearity analysis

Based on SSR polymorphic markers and CRI-SNP markers, a linkage map of chr25 harboring 210 loci was constructed. The map covers a total distance of 178.8 cM, with an average distance between markers of 0.85 cM (S1 Fig) and no gap larger than 10.0 cM. The largest two adjacent-marker gaps larger than 5.0 cM were located at each end of the chromosome. One gap was between markers SHH90056 (at position 1.9 cM) and SWU19721 (7.0 cM), separated by a distance of 5.1 cM; the other was between markers CRI-SNP140898 (166.3 cM) and SWU19008 (175.7 cM), separated by a distance of 9.4 cM. Only a few minor gaps were detected across the entire chromosome, mainly in the upper part of the linkage map upstream of SWU19607 (25.3 cM) and the lower part downstream of CRI-SNP112817 (124.7cM).

**Fig 2:**
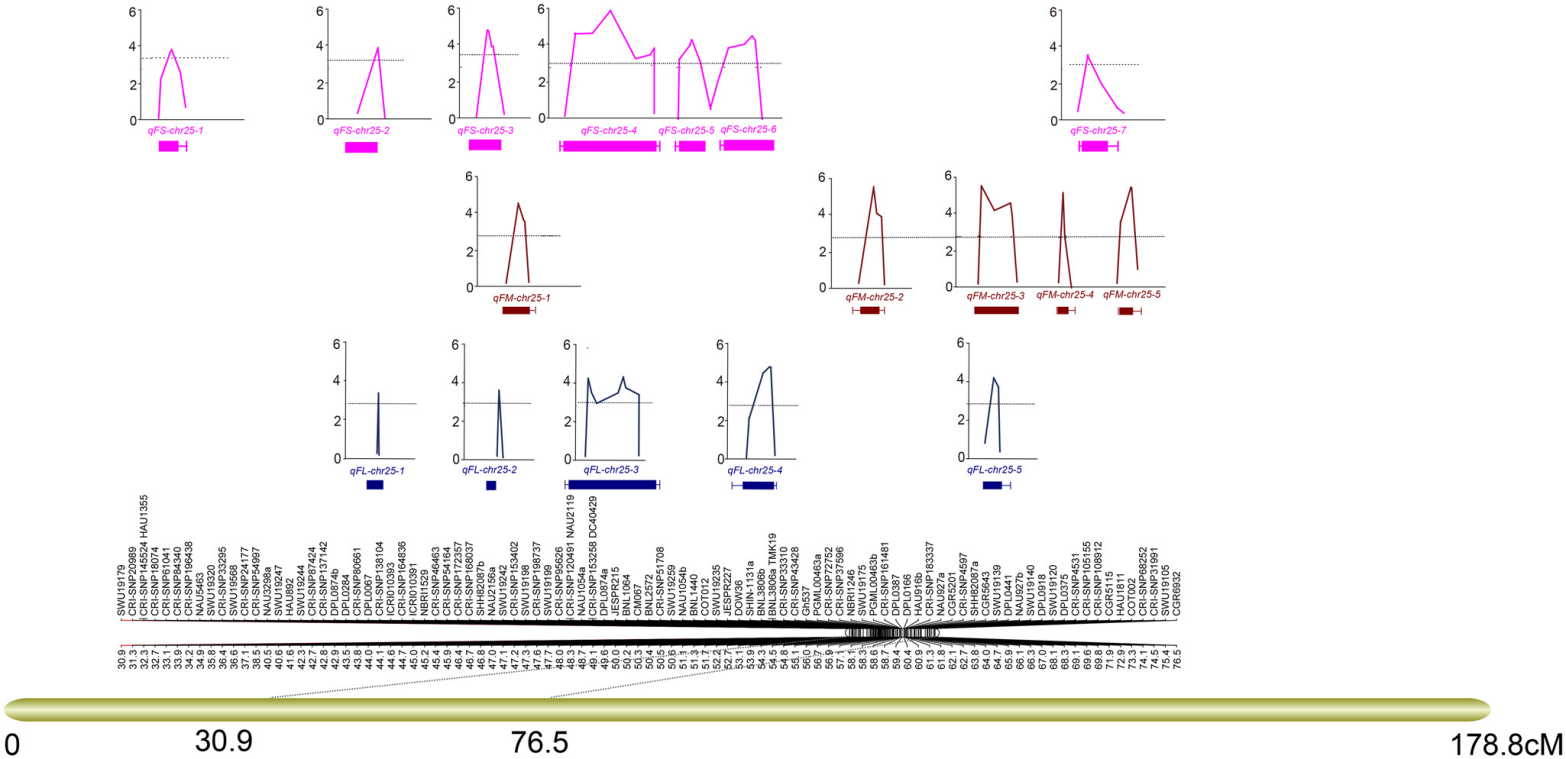
QTL mapping of fiber length, fiber micronaire and fiber strength. A zigzag line indicates a QTL and the top point of the zigzag line indicates the QTL position. The dotted line indicates the threshold of a QTL. The box-and whisker diagram indicates the range of a QTL.

The map also consisted of a region that harbored a high resolution map of markers from marker SWU19244 (42.27 cM) to DPL0918 (66.97 cM) with an average marker distance of 0.35 cM and no visible marker gaps. The major QTLs detected in the present study were also located within this region (Fig 2 and S1 Fig).

**Fig 3:**
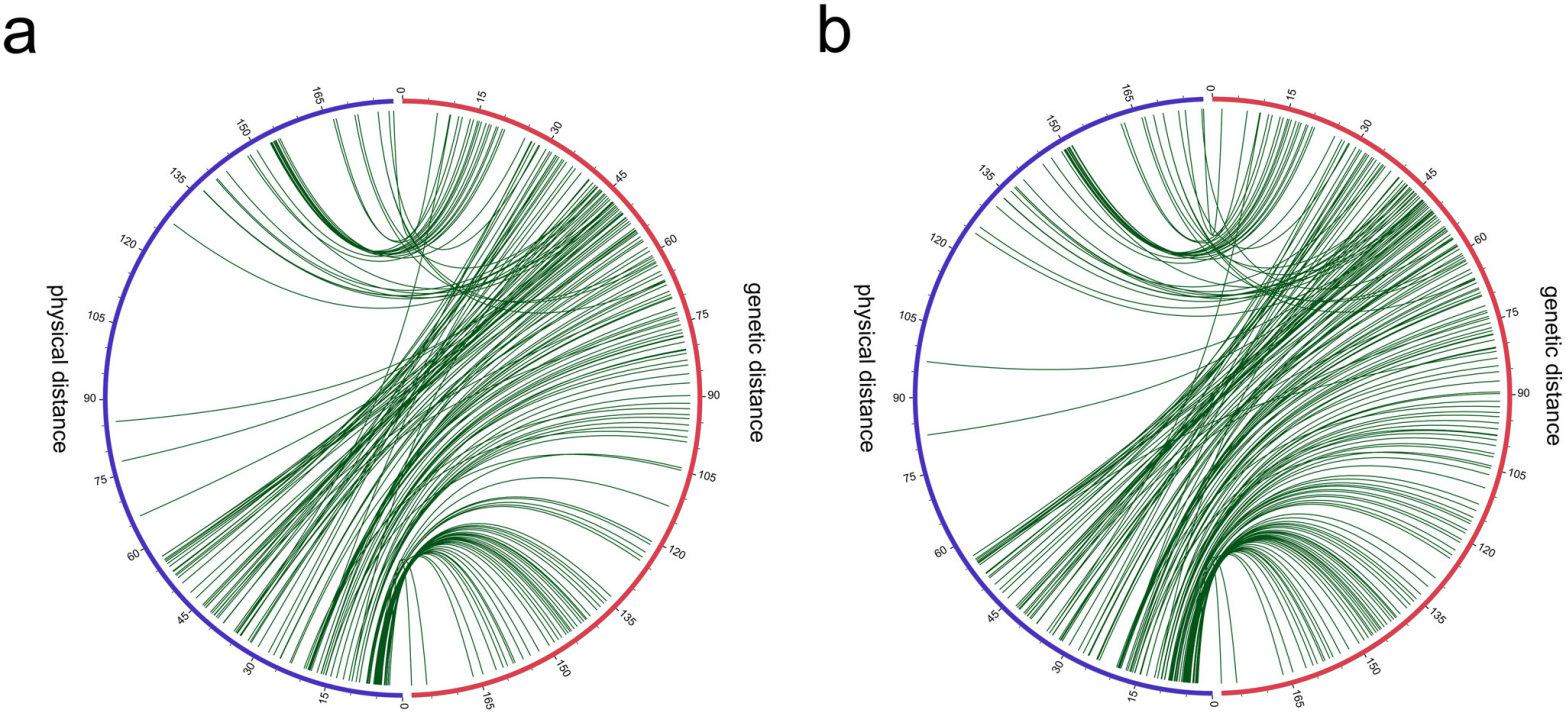
Collinearity of marker positions in the genetic and physical maps using the genome of a: *Gossypium hirsutum*; and b: *Gossypium raimondii*.

The result of the collinearity analysis between the linkage map and the corresponding physical map of *G*.*hirsutum*[46, 47] and *G*. *raimondii* [26, 27] are shown in Fig 3(also see S4 Table). Most of the positions of the CRI-SNP and SSR markers showed good fitness between the two maps, whereas a few of the CRI-SNP markers were aligned into the scaffold of chrr25 of *G*.*hirsutum* genome (S4 Table). These results suggested that the linkage map was reliable for further QTL mapping.

### QTL mapping of fiber strength, fiber length and FM in the RIL population

A total of 37 QTLs for fiber quality traits (fiber strength, fiber length and FM) were detected on chr25 in the RILs across 11 environments. Seventeen of these could be detected in at least two environments explaining 5.17%–11.83% of the total observed PV, among which seven were for fiber strength, five for fiber length, and five for FM (Table 3 and Fig 2). The remaining 20 QTLs were detected only in one environment, explaining as much as 11.69% of the total observed PV (S5 Table).

**Table 3:** Stable QTLs for fiber length, fiber micronaire and fiber strength identified in the RILs.

Trait	QTL	Environment	Marker interval	Position	LOD	Additive	R² (%)
FL	qFL-chr25-1	09QZ	DPL0067-CRI-SNP138104	44.01	3.03	0.50	10.81
		10AY		44.01	3.96	0.53	8.22
	qFL-chr25-2	10GY	NAU2156a	47.01	3.37	0.48	7.04
		10AY		47.01	3.03	0.46	6.54
	qFL-chr25-3	10AY	CRI-SNP120941(NAU2119)-BNL2572	48.31	4.49	0.55	9.32
		13AY		48.31	3.01	0.34	6.21
		09QZ		49.71	4.85	0.50	10.27
		10GY		49.71	3.16	0.46	6.47
	qFL-chr25-4	10GY	DOW036-TMK19(BNL3806a)	54.01	3.71	0.50	7.47
		09AY		54.51	3.75	0.46	7.17
		09QZ		54.51	5.33	0.56	11.23
		10AY		54.51	4.66	0.61	9.58
	qFL-chr-25-5	09AY	CGR5463-DPL0441	64.01	3.71	0.46	8.00
		09AKS		64.01	3.20	0.63	7.07
FM	qFM-chr25-1	08AY	SWU19242-SWU19198	47.11	2.64	-0.17	5.65
		09AKS		47.21	2.27	-0.14	8.24
		13AY		47.31	2.68	-0.16	6.21
	qFM-chr25-2	08AY	NBRI1246-PGML00463b	58.11	3.13	-0.13	6.46
		08LQ		58.11	5.36	-0.17	11.75
		08QZ		58.11	2.89	-0.13	6.07
		13AY		58.11	3.02	-0.23	6.89
	qFM-chr25-3	08LQ	SHH82087a-DPL0441	64.01	5.46	-0.18	11.09
		08QZ		64.01	3.44	-0.15	6.95
		09QZ		64.01	3.57	-0.16	7.33
		10GY		64.01	3.41	-0.15	6.80
		10ZZ		64.81	3.42	-0.16	6.92
	qFM-chr25-4	08LQ	SWU19120-DPL0375	68.11	4.95	-0.17	10.28
		09QZ		68.31	3.07	-0.17	6.49
	qFM-chr25-5	10GY	COT002-CRI-CRI-SNP68652	73.31	3.92	-0.16	8.04
		10ZZ		73.31	2.95	-0.16	6.25
FS	qFS-chr25-1	08LQ	CRI-SNP61041-CRI-SNP196438	33.91	2.78	0.65	5.17
		10ZZ		33.91	2.77	0.56	5.72
	qFS-chr25-2	09AKS	CRI-SNP80661-CRI-SNP138104	44.01	3.24	0.63	7.28
		10AY		44.01	3.21	0.77	8.40
	qFS-chr25-3	09AKS	SHH82078b-SWU19242	47.01	4.72	0.72	8.76
		13AY		47.01	4.08	0.87	8.52
	qFS-chr25-4	09AY	CRI-SNP120491(NAU2119)-CRI-SNP51708	48.31	3.52	0.73	6.53
		10GY		48.31	3.77	0.61	7.28
		10AY		48.71	4.83	0.86	9.74
		07AY		49.11	3.54	0.70	7.06
		09QZ		49.11	5.37	0.91	11.83
		09AKS		49.11	6.57	0.81	11.69
		13AY		50.21	4.83	0.91	9.62
	qFS-chr25-5	10AY	NAU1054b-COT012	51.11	4.18	0.78	8.39
		09AKS		51.31	4.69	0.69	8.61
	qFS-chr25-6	10GY	JESPR227-TMK19(BNL3806a)	54.01	3.77	0.62	7.29
		09AY		54.41	3.76	0.76	7.03
		09AKS		54.41	4.57	0.72	8.25
	qFS-chr25-7	07AY	CRI-SNP105155-HAU1811	69.81	2.73	0.62	5.60
		08LQ		70.81	3.03	0.75	7.07
		10GY		70.81	2.86	0.64	6.90

For fiber strength, the details of the seven QTL identified were as follows: qFS-chr25-4 was detected in seven environments, was located within the interval between markers CRI-SNP120491 and CRI-SNP51708, and could explain 6.53%–11.83% of the observed PV. qFS-chr25-6 and qFS-chr25-7 were detected in three environments, were located in the intervals between markers JESPR227 and BNL3806a, and between CRI-SNP105115 and HAU1811, and explained 7.03%–8.25% and 5.60%–7.07% of the observed PV, respectively. qFS-chr25-1, qFS-chr25-2, qFS-chr25-3 and qFS-chr25-5 were detected in two environments, were located in the intervals between markers CRI-SNP61041 and CRI-SNP196438, CRI-SNP80661 and CRI-SNP138104, SHH82078b and SWU19242, and NAU1054b and COT012, and explained 5.17%–5.72%, 7.28%–8.40%, 8.52%–8.76% and 8.39%–8.61% of the observed PV, respectively.

For fiber length, the details of the five identified QTLs were: qFL-chr25-3 and qFL-chr25-4 were detected in four environments, were located in the intervals between markers CRI-SNP120941 and BNL2572, and DOW036 and TMK19, and explained 6.21%–10.27% and 7.17%–11.23% of the observed PV. qFL-chr25-1, qFL-chr25-2 and qFL-chr25-5 were detected in two environments, were located in the intervals between DPL0067 and CRI-SNP138104, at NAU2156a, and between CGR5463 and DPL0441, and explained 8.22%–10.81%, 6.54%–7.04% and 7.07%–8.00% of the observed PV.

For FM, the details of the five identified QTLs were: qFM-chr25-3 was detected in five environments, was located in the marker interval between SHH82087a and DPL0441, and explained 6.80%–11.09% of the observed PV. qFM-chr25-2 was detected in four environments, was located in the interval between markers NBRI1246 and PGML00463b, and explained 6.07%–11.75% of the observed PV. qFM-chr25-1 was detected in three environments, was located in the interval between markers SWU19242 and SWU19198, and explained 5.65%–8.24% of the observed PV. qFM-chr25-4 and qFM-chr25-5 were detected in two environments, were located in the intervals between markers SWU19120 and DPL0375 and between COT002 and CRI-SNP68652, and explained 6.49%–10.28% and 6.25%–8.04% of the observed PV, respectively.

### Meta-analysis of QTLs

**Fig 4:**
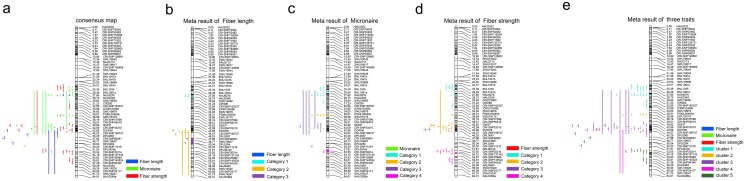
Meta analysis of QTLs for fiber length, fiber micronaire and fiber strength. a: concensus map; b: meta result of fiber length; c: meta result of fiber micronaire; d: meta result of fiber strength; e: meta result of all the three traits.

Three maps [20, 23] including the current map (Fig 4) were used in the construction of a consensus map and in QTL meta-analysis. A consensus map consisting of 55 markers spanning a linkage distance of 64.01 cM and average marker interval of 1.19 cM was generated (Fig 4). Meta-analysis indicated that the QTLs of fiber length, FM and fiber strength aggregated into three, four, and four categories respectively. Fiber length and FM each consisted of a category that comprised only two QTLs (Fig 4b and 4c). For fiber length, the three categories consisted of two, six and two QTLs and aggregated at linkage regions 28.75–29.94 cM, 44.07–45.25 cM and 47.66–50.20 cM respectively. For FM, the four categories consisted of nine, four, ten and two QTLs and aggregated at linkage regions 25.39–32.65 cM, 36.98–38.19 cM, 42.09–44.07 cM and 50.20–54.15 cM respectively. For fiber strength, the four categories consisted five, eleven, five and ten QTLs and aggregated at the linkage regions 25.39–33.89 cM, 36.98–44.07 cM, 44.07–45.25 cM, and 51.81–58.38 cM respectively. Clusters were also aggregated among the three fiber traits (Fig 4e). Category 1 of fiber length, category 1 of micronaire and category 1 of fiber strength were aggregated into cluster 1, category 2 of micronaire and a few QTLs of fiber strength were aggregated into cluster 2, category 3 of micronaire and category 2 of fiber strength were aggregated into cluster 3, category 2 of fiber length and category 3 of fiber strength were aggregated into cluster 4 and finally category 3 of fiber length, category 4 of Micronaire, and category 4 of fiber strength were aggregated into cluster 5.

### Expressions of candidate genes Gh_D06G0694, Gh_D06G0721, Gh_D06G0740 and Gh_D06G1025

**Fig 5:**
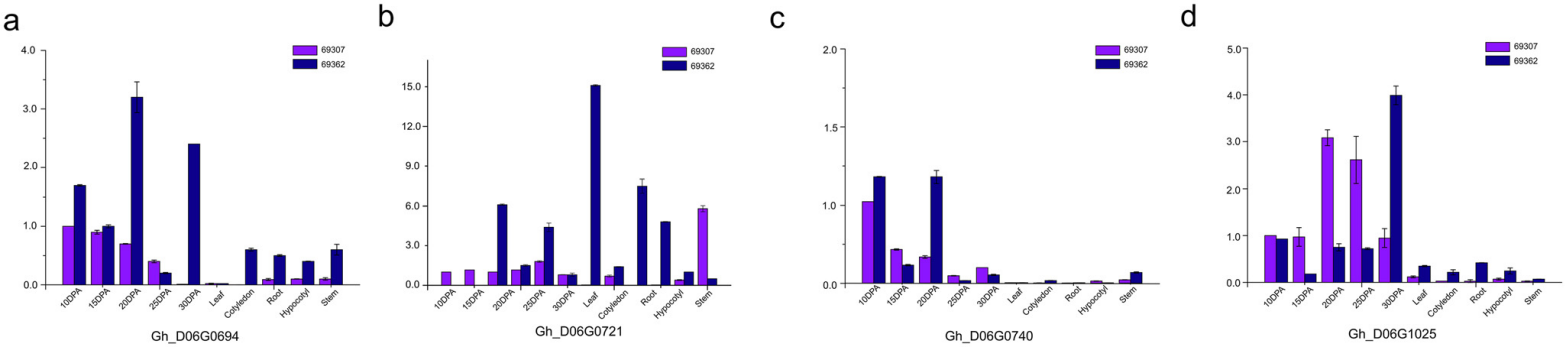
Expressions of candidate genes Gh_D06G0694, Gh_D06G0721, Gh_D06G0740 and Gh_D06G1025. a: Expression of Gh_D06G0694; b: Expression of Gh_D06G0721; c: Expression of Gh_D06G0740; d: Expression of Gh_D06G1025.

The expression patterns of the candidate genes across various developmental stages of cotton fiber and plant cells were shown in Fig 5. except for Gh_D06G0721, which did not show a specific expression in cotton fiber cells, all the other three candidate genes, Gh_D06G0694, Gh_D06G0740 and Gh_D06G1025, showed specific and differential expression patterns in developing fiber cells between the two lines 69307 and 69362. For Gh_D06G0694, its expression in 69307 at 10dpa was the highest and then gradually decreased until 25dpa, whereas its expression in 69362 was highest at 20 dpa and was sustained until 30dpa. For Gh_D06G0740, its expression in 69307 at 10dpa was also the highest and then gradually decreased until 30 dpa, whereas in 69362, the highest expression levels were only observed at 10 dpa and 20 dpa. For Gh_D06G1025, its expression in 69307 increased from 10 dpa to 20 dpa and then descrased until after 30 dpa, whereas its expression in 69362 remained at an intermediate level from 10 dpa to 25 dpa then increased by 30 dpa.

## Discussion

### Genetic map construction

Of all the SSR markers mapped in the current linkage map, the SWU primer pairs were the most recently developed and reported by Tang et al. [21] Most of the other SSR primer pair sets have been frequently used in genetic map construction [2, 19, 21,48,49]. Their low rates of polymorphism indicated that these linkage maps generally did not have a sufficient coverage of the entire genome, thus presented gaps or showed two or more linkage groups within one chromosome [14,18,20,21]. Shi et al. [50] employed an inter-specific BC_1_F_1_ population of *G*. *barbadense* L. and *G*. *hirsutum* L.to constructed a genetic map in which chr25 harbored 79 SSR markers, covering a total distance of 160.80 cM, with an adjacent-marker interval of more than 2 cM. Yu et al. [24] used an inter-specific F_2_ population between *G*. *barbadense* L. and *G*. *hirsutum L*. to construct a genetic map that harbored only 36 markers in chr25, with an avenge adjacent-marker distance of more than 3.00 cM. Shao et al. [20] used three different populations to construct genetic maps, in which the maps of chr25 comprised two linkage groups, the largest one of which harbored only 20 markers. Tang et al. [21] constructed a comparable integrative map of chr25 that harbored 55 markers with an average marker interval of 1.70 cM. Similar results were also obtained by Liang et al. [18], Fang et al. [22] and Yang et al. [25]. Other reports that constructed maps did not detect any markers on chr25 [19,44,49]. The map produced in the present study is much more saturated, harboring SNP loci on the basis of SSR markers, which provided a solid prerequisite for QTL identification and mapping.

### QTL mapping and their potential applications

A total thirty-seven QTLs were identified for fiber length, fiber strength and micronaire on chr25, 17 of which were stably expressed in at least in two environments. The SNPs identified in present study existed in hot spots and clusters. The seven QTLs for fiber strength covered a genetic distance of 39.80 cM, encompassing 33.10 cM to 72.90 cM, whereas three of these (qFS-chr25-4, qFS-chr25-5 and qFS-chr25-6) were detected only within a short genetic distance of 6.20 cM. Three of the five QTLs for fiber length (qFL-chr25-1, qFL-chr25-2 and qFL-chr25-3) were detected at a genetic distance of only 10.30 cM, and three of the five QTLs for micronaire encompassed 9.50 cM (Fig 2). qFL-chr25-1 and qFS-chr25-2, and qFL-chr25-5 and qFM-chr25-3 shared the same right flanking markers, whereas qFL-chr25-3, qFL-chr25-4, qFS-chr25-4, qFS-chr25-5 and qFS-chr25-6 were tightly linked and shared overlapping marker regions. qFS-chr25-3 and qFL-chr25-2 shared the same markers region and qFM-chr25-1 was tightly linked to that region. The linkage of these QTLs also reflected the agronomic behavior of fiber strength, fiber length and FM in the field assessment. The results indicated that the QTLs conferring different fiber quality traits clustered in genetic regions on chr25, encompassing the region from 33.10 cM to 74.10 cM. Future breeding practice using MAS should pay more attention to such spots and clusters.

QTLs on chr25 conferring fiber quality traits were also identified in previous studies [20,23,25]. Sun et al. [23] identified two QTLs for fiber strength, fiber length and micronaire,. The two QTLs for fiber strength could be the same as qFS-chr25-4 and qFS-chr25-5; however, we were unable to differentiate these because the two flanking markers of one of Sun’s fiber strength QTLs were located separately in the regions of qFS-chr25-4 and qFS-chr25-5, respectively, and the other two flanking markers of Sun’s second fiber strength QTL were also located separately in regionqFS-chr25-4 and in an irrelevant position. Sun et al [23] also identified two fiber length QTLs on chr25; however, because the map on which the QTLs were identified was not saturated and integrative, the QTLs they identified spanned a large gap. In almost the same region, we identified five QTLs for fiber length. Similar results were obtained for the identification of QTLs for FM. Shao et al. [20], using three upland cotton populations, identified two QTLs for fiber strength and one for fm on chr25. Because there are no shared markers and there are at least two short linkage groups in each map, it is difficult to identify their counterpart QTLs in the present study. Yang et al. [25] identified two QTLs for FMusing an interspecific BC_1_ population between *G*. *hirsutum* and *G*. *barbadense*, which covers the short linkage group.

Biomercator V4.2 is a useful tool for comparing QTLs from different studies and the detection of QTL hotspots and clusters [20, 23]. There were two groups of data used in QTL meta-analysis: previously published QTL data [20,23] and the current QTL data on the 11 environments. As the previous maps did not involve the entire chromosome consisted of gaps and was less saturated, the QTLs often had large confidence intervals that indicate QTL identification with less precision. [Fig 4]. Category 2 of fiber length [Fig 4b], category 3 of micronaire[Fig 4c] and category 2 of fiber strength[Fig 4d] contained QTLs generated from the present study and those from previous investigations. All the rest of the categories solely contained ours results. As the QTLs identified in our study encompassed multiple environments, the QTLs of each trait aggregated into one category and thus could be regarded as stable QTLs for the corresponding environment. Therefore, one stable QTL for each of the three traits identified in the present study was also reported in previous studies, whereas two stable QTLs of fiber length, three stable QTLs of micronaire and three stable QTLs of fiber strength were identified by the present study on chr25 based on a saturated and integrated genetic map. Our results also indicated that using a saturated high-resolution map allowed the identification of a higher number of QTLs and the QTLs could be mapped more precisely and to smaller regions, which will greatly increase its utility in breeding programs.

The utility of a QTL is determined by two aspects: the percentage of the observed PV that the QTL can explain and whether the QTL is expressed stably across multiple environments [20]. Of the 17 multi-environment QTLs in the present study, nine were expressed in two environments, namely qFS-chr25-1, qFS-chr25-2, qFS-chr25-3, and qFS-chr25-5 for fiber strength; qFL-chr25-1, qFL-chr25-2 and qFL-chr25-5 for fiber length; and qFM-chr25-4 and qFS-chr25-1 for FM. Three QTLs were stably expressed in three environments (qFS-chr25-6 and qFS-chr25-7 for fiber strength and qFM-chr25-1 for FM. Two QTLs were stably expressed in four environments: qFL-chr25-3 and qFL-chr25-4 for fiber length. One QTL was expressed stably in five environments and one in seven environments: qFM-chr25-3 and qFS-chr25-4, respectively. These QTLs will be valuable in breeding programs for cotton fiber improvement. In addition, there were several QTLs that were only expressed in one environment (S5 Table). We considered these as unstable QTLs, however, one of them could explain as much as 11.69% of the observed PV, (S5 Table). Therefor these unstable QTLs may be also important, to some extent, in the deveploment of cultivars with superior fiber quality.

To further study genetic mapping and QTL identification, QTL analysis of a candidate gene based on fine mapping is warranted. Several candidate genes were pooled from Zhang et al [46] and tested for their expression profiles. Except that the gene Gh_D06G0721 did not show specific expression in fiber cells, the other three gene showed specific expression in fiber cells and their expression profiles between 69307 and 69362 were different. Gene ontology (GO) analysis indicates that Gh_D06G0694 is related to protein transportation, Gh_D06G0740 is related to hydrolase activity, and Gh_D06G1025 is related to protein binding or protein homooligomerization. Whether they contribute to the second wall thickening of fiber cells or not still need further studies.

## Supporting Information

S1 Table The sequence of the SSR markers on the map. (XLSX)

S2 Table The sequence of the SNP markers on the map. (XLSX)

S3 Table Correlation analyses between fiber quality traits based on mean values of eleven environments. (XLSX)

S4 Table Collinearity of markers position in genetic linkage and physical maps of *Gossypium raimondii* and *Gossypium hirsutum*. (XLSX)

S5 Table Mapping of the QTL that only identified in one environment. (XLSX)

S1 Fig Genetic mapping and major QTL mapping of chromosome 25. (TIF)
