# Supplementary figures and images for: High Resolution Consensus Mapping of Quantitative Trait Loci for Fiber Strength, Length and Micronaire on Chromosome 25 of the Upland Cotton (Gossypium hirsutum L.)
Source: PLoS One. 2015 Aug 11;10(8):e0135430. doi: 10.1371/journal.pone.0135430 (PMC4532425; doi:10.1371/journal.pone.0135430)

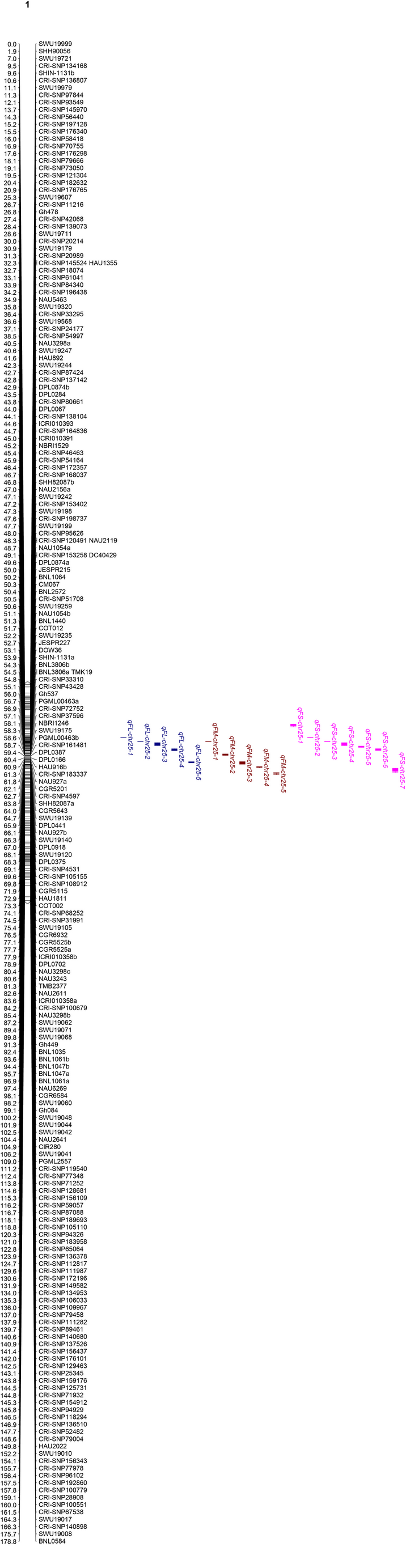

Supplement: S1 Fig — (TIF) [file pone.0135430.s006.tif]
